# Tinea capitis caused by *Trichophyton violaceum* in an immunocompetent elderly patient: A case report and review of literature

**DOI:** 10.1002/ccr3.8205

**Published:** 2023-11-20

**Authors:** Mehdi Gheisari, Khatere Zahedi, Nabaa Al‐Zubaidi

**Affiliations:** ^1^ Skin Research Center Shahid Beheshti University of Medical Sciences Tehran Iran; ^2^ Department of Dermatology, Loghman Hakim Hopsital Shahid Beheshti University of Medical Sciences Tehran Iran

**Keywords:** dermatophytosis, elderly, tinea capitis, trichophyton

## Abstract

Tinea capitis is a common dermatophyte infection of the scalp in children. It is an uncommon infection in adults and usually affects postmenopausal women and immunocompromised patients. We report an immunocompetent elderly female with inflammatory tinea capitis caused by *Trichophyton violaceum* and review the literature for the past 5 years to describe the disease, its epidemiologic characteristics, dermatophyte species involved and treatment options used. The total number of cases was 11, including 8 women and 3 men, with an average age of 48.36. The most commonly isolated dermatophyte was *Trichophyton tonsurans*, and most cases were treated successfully with oral terbinafine with no side effects. In our case, the diagnosis was established by direct examination, culture and histological examination. Remedy with itraconazole and prednisolone was very successful. Early diagnosis of tinea capitis in adults is necessary to provide early treatment and minimize sequelae of the disease.

## Graphical abstract

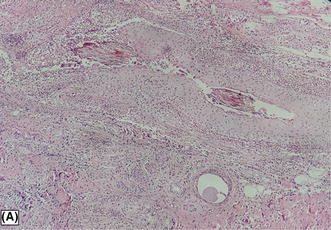

## 1 INTRODUCTION

Tinea capitis is a common infection of the scalp and hair caused by dermatophyte fungi that principally affects children.^1^ It is an uncommon infection in adults and generally occurs in postmenopausal women and immunocompromised patients, e.g., AIDS patients, transplant recipients, patients receiving high‐dose steroid therapy, and patients with diabetes mellitus.^2^, ^3^ Adult tinea capitis may have atypical clinical presentations.^2^, ^3^, ^4^ The causative pathogens in children and adults belong to two genera: *Trichophyton* and *Microsporum*.^5^ The clinical manifestations are characterized by an erythematous and scaly plaques, itching, suppurative swelling with purulent discharge, areas of alopecia, and regional lymphadenopathy. It is often misdiagnosed as a bacterial infection, leading to unnecessary antibiotic prescription or surgical intervention. Treatment delay may result in permanent hair loss.^6^, ^7^ The diagnosis of tinea capitis is made by fungal culture (gold standard), microscopy, wood's lamp, and trichoscopy.^5^, ^8^

## 2 CASE REPORT

**FIGURE 1:**
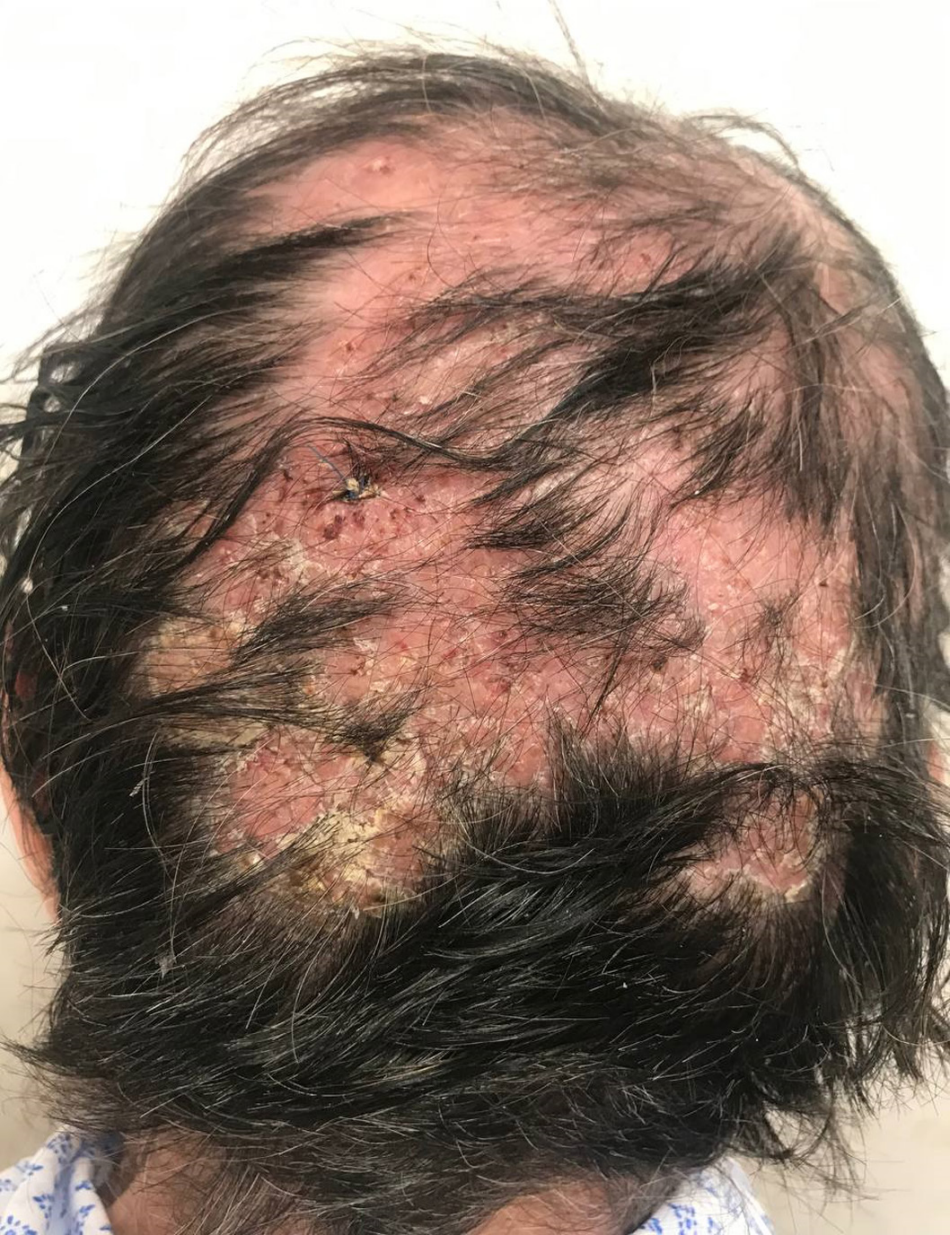
Erythematous papules and plaques over the scalp of an elderly female, with yellow crust, pustule formation, and hair loss.

**FIGURE 2:**
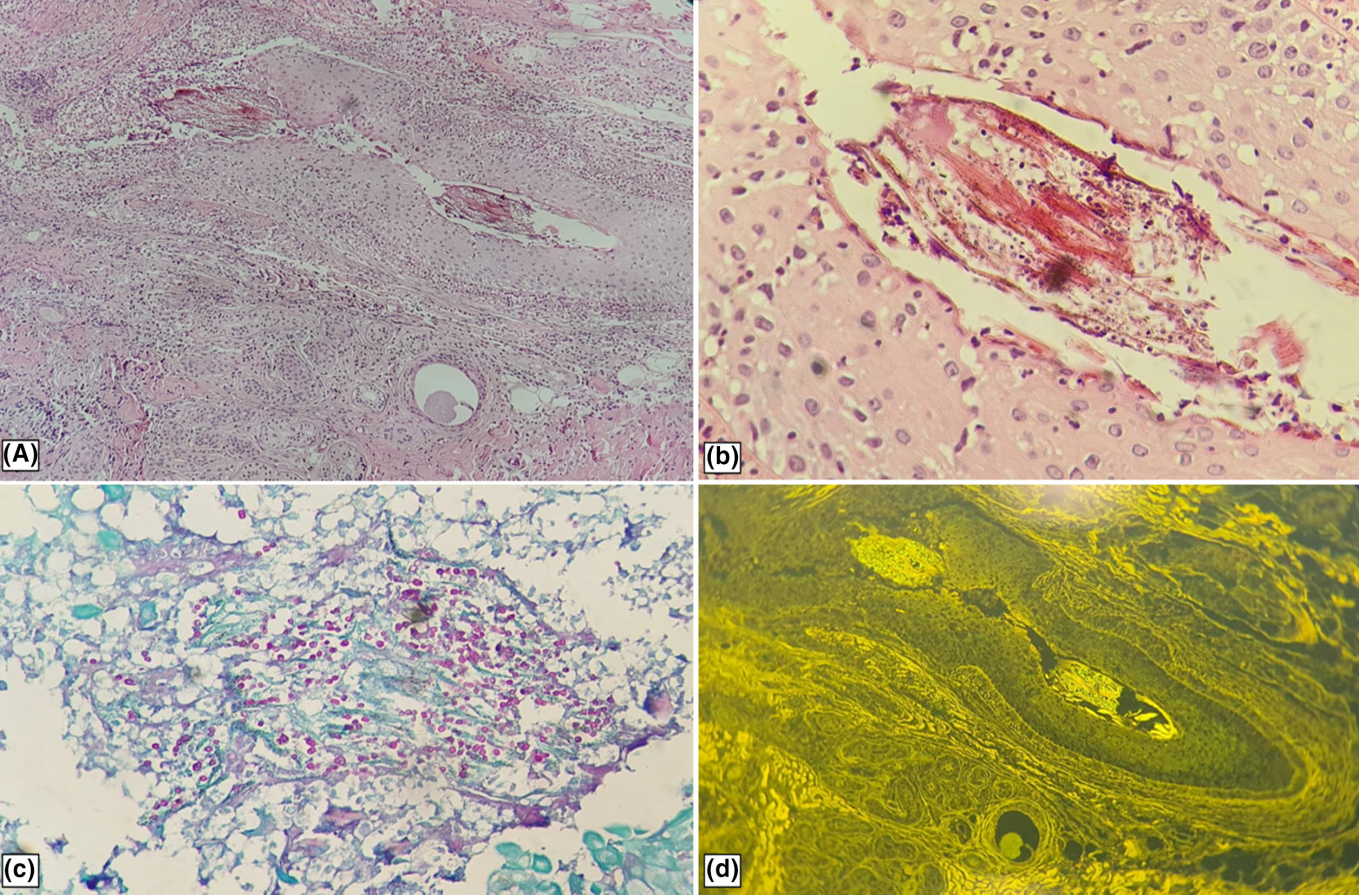
Tinea capitis. (A and B) Septate hyphae and spores invading the hair shaft of disrupted hair follicle (hematoxylin and eosin staining, ×100 (A) and ×400 (B)). (C) Endothrix septate hyphae invading the hair shaft (PAS staining, ×400). (D) Endothrix hair infection by green fluorescent septate hyphae and spores (Fluorescent microscope, ×100).

**FIGURE 3:**
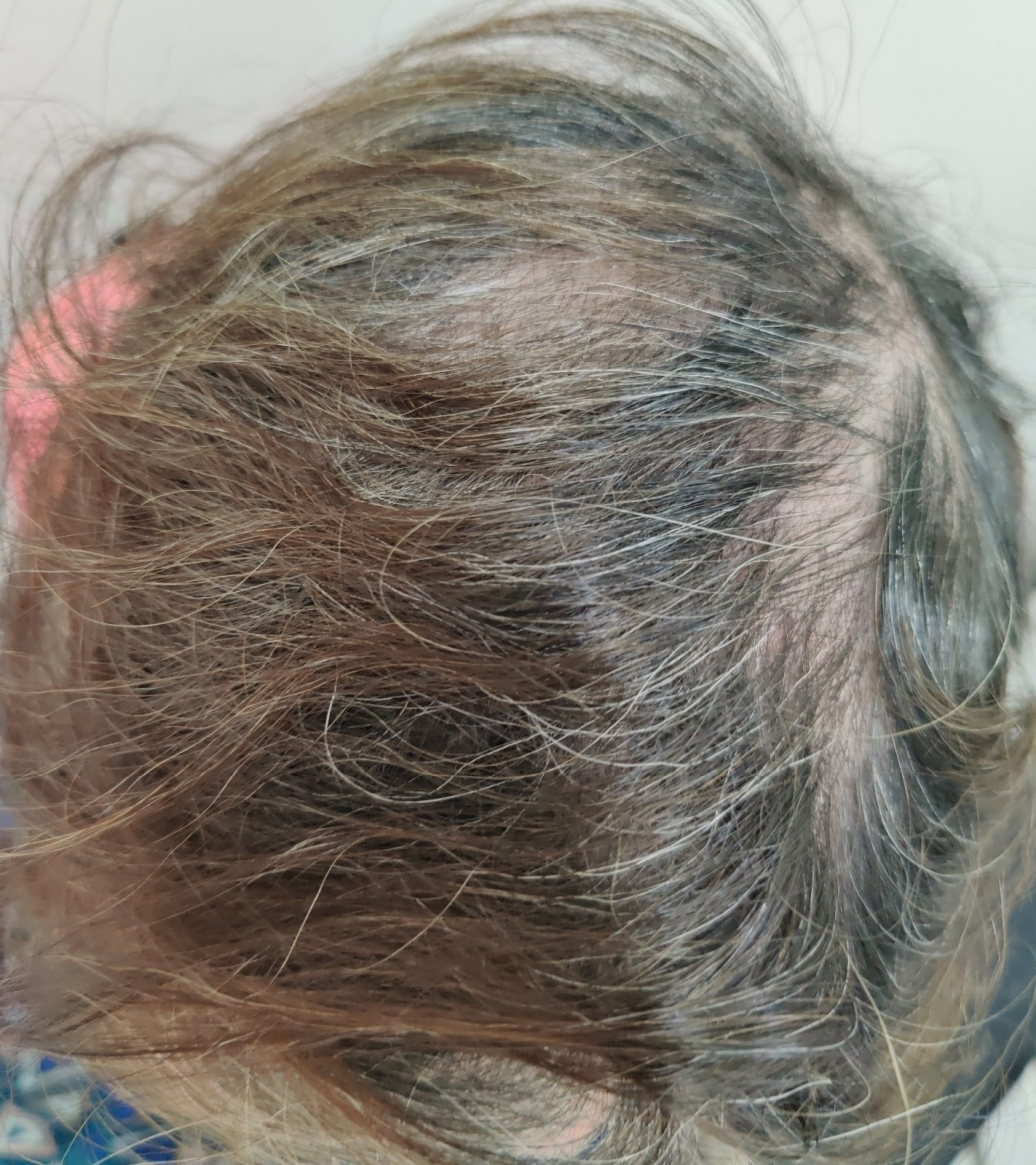
Hair regrowth and complete clearance of all inflammatory lesions.

A 75‐year‐old female presented with a 3‐month history of pruritic, purulent and crusted lesions over the scalp. She had been treated with multiple oral antibiotics and a topical cream consisting of clobetasol and salicylic acid for 1 month at another clinic, which worsened the patients' symptoms. The patient had no medical history other than hypertension. She was in a good general condition and had not received any immunosuppressant drug. There was not any similar disease in other family members. Physical examination showed multiple erythemato‐edemathous papules and plaques with yellow crust, pustule formation, and hair loss involving the vertex and occipital area of the scalp (Figure 1). There were no other lesions in any other parts of the skin, nails, and mucosa. Laboratory examination revealed a: fasting blood sugar (FBS) = 94 mg/dL, urea = 44 mg/dL, serum creatinine (SCr) = 0.9 mg/dL, serum glutamic‐oxaloacetic transaminase (SGOT) = 23 IU/dL, serum glutamic pyruvic transaminase (SGPT) = 19 IU/dL, alkaline phosphatase (ALP) = 121 U/dL, erythrocyte sedimentation rate (ESR) = 37 mm/h, and C‐reactive protein (CRP) = 0.6 mg/dL. The complete blood count (CBC) and the serum electrolyte tests were normal. The viral serology and the interferon‐g release assay (IGRA) were negative. The clinical differential diagnosis included pemphigus vulgaris, folliculitis decalvans, erosive pustular dermatosis, and tinea capitis. First, we performed a dermoscopy, and the presence of “comma”, “corkscrew,” and dystrophic broken hairs was the clue for the diagnosis of tinea capitis. Then, according to the results of the dermoscopy, we performed a KOH smear and culture. The direct exam with 20% KOH showed an endothrix infection, and the mycological culture showed the growth of *Trichophyton violaceum*. Bacterial culture was negative. Skin biopsy of the scalp lesions showed an acute superficial and deep folliculitis with intrafollicular mycelial fungal infection consistent with tinea capitis (endothrix), on hematoxylin and eosin staining (Figure 2A,B). PAS‐stained slides showed endothrix septate hyphae invading the hair shafts (Figure 2C). Fluorescent microscopy showed endothrix infection by green fluorescent, septate hyphae, and spores (Figure 2D). The patient was treated with prednisolone 15 mg daily for 1 month and oral itraconazole 400 mg daily, which was gradually tapered to 100 mg daily at the last 2 months. The liver and renal function tests were evaluated regularly during the treatment period, and all were in the normal ranges. Also, the patient and all family members were treated with 2.5% selenium sulfide shampoo. There was complete clearance of the lesions and acceptable hair regrowth (Figure 3).

## 3 DISCUSSION

The amount of fungistatic saturated fatty acids in sebum increases at puberty and therefore dermatophyte colonization of the scalp disappears in this age.^9^ This is thought to explain the rarity of tinea capitis in adults. Although the disease was once thought to be rare in adulthood, studies have been increasingly reporting tinea capitis among adults especially in immunocompromised patients, menopausal, and elderly women.^2^, ^3^, ^4^ Our patient was a 75‐year‐old menopause female, but not immunocompromised. In most of the reported cases, including our case, the diagnosis was delayed. This delay is probably due to both the rarity of this infection in adults and its atypical clinical presentation. The disease may resemble bacterial folliculitis, folliculitis decalvans, dissecting cellulitis, pityriasis amiantacea and its related etiologies, and scaring alopecia like lupus erythematosus.^10^ In many studies, the correct diagnoses were established by tissue culture.^3^, ^4^ Although, for some authors, griseofulvin remains the treatment of choice for tinea capitis in children and adults, both terbinafine and itraconazole are considered acceptable alternatives.^2^, ^3^, ^4^ Due to the numerous reports describing treatment‐resistant dermatophytosis, which has emerged as a global public health threat,^11^, ^12^, ^13^, ^14^ we started the treatment with high‐dose itraconazole as 400 mg daily. Also, we prescribed prednisone 15 mg daily at the first month because of the severe inflammation. Our patient responded well to this treatment, and there was complete clearance of the lesions with acceptable hair regrowth.

We reviewed tinea capitis case reports in adults indexed in PubMed between 2018 and 2023. To be included in the review, articles had to be available in the English language. Inclusion criteria included patient age ≥18 years, diagnosis of tinea capitis, no history of immunosuppression or receiving any immunosuppressant drugs, no history of other medical conditions or history of other dermatophytosis infection in other parts of the skin, no history of gardening, pet‐keeping, contact with domestic animals, or other individuals with the same manifestations or dermatophytosis infection, and no history of contact with objects containing fomites, including brushes, combs, bedding, clothing, toys, furniture, and telephones (Table 1).

**TABLE 1:** Tinea capitis case reports in adults from 2018 to 2023.

Authors	Year	No.	Age	Sex	Dermatophyte isolated	Disseminated lesions	Rx
Ngan Do et al^15^	2020	1	55 years	M	Trichophyton rubrum	+	Oral terbinafine for 3 months
Saber Dooqaei Moqadam et al^18^	2020	2	39 years	F	Endothrix tinea capitis/no culture		Oral terbinafine 250 mg daily for 8 weeks and 2% ketoconazole shampoo
Debora Semeia Takaliuang et al^19^	2022	3	19 years	F	Endothrix tinea capitis/no culture		Oral griseofulvin 500 mg BD for 6–8 weeks, cetirizine tablets daily, ketoconazole shampoo 2% TDS for 2 weeks
Yoshihiro GOTO et al^20^	2019	4	18 years	M	*Trichophyton tonsurans*		Oral terbinafine 250 mg daily
Francisca ALVES et al^21^	2019	5	92 years	F	*Microscoporum audouinii*		The patient died
Yu‐Shi Zheng et al^16^	2020	6	53 years	F	*Trichophyton tonsurans*		Oral terbinafine (0.25 g/day) and naftifine hydrochloride and ketoconazole cream BD for 2 weeks
Michela Starace et al^22^	2022	7	66 years	F	*Trichophyton tonsurans*		Oral terbinafine, oral corticosteroids, and topical ciclopirox and naftifine creams for 3 months
Yanyan Zhu et al^17^	2021	8	64 years	F	*Trichophyton violaceum*	+	Oral itraconazole 200 mg BD for 3 months ketoconazole cream BD
Enzo Errichetti et al^23^	2021	9	37 years	M	*Microsporum Canis*		Oral terbinafine 250 mg daily for 8 weeks
Faten Rabhi et al^24^	2023	10	29 years	F	*Trichophyton violaceum*		Oral terbinafine
Anuja Yadav et al^25^	2020	11	60 years	F	Endothrix tinea capitis/no culture		Oral terbinafine 250 mg daily and 2% ketoconazole shampoo for 6 weeks

We found a total of 11 cases. Of these cases, the prevalence was higher in women (8/11) and the average age was 48.36. Three cases did not have a mycological culture and did not mention the dermatophyte isolated. *Trichophyton tonsurans* was the most common dermatophyte, followed by *Trichophyton violaceum*. Most cases were treated with oral terbinafine 250 mg daily. One patient was treated with oral griseofulvin 500 mg every 12 h and another one with oral itraconazole 200 mg twice daily. Most patients received combination therapy consisting of oral and topical antifungal agents. All patients reported were cured successfully without any side effects. Two cases had disseminated lesions on the face,^15^ extremities, and nails^15^, ^17^ years after the scalp manifestations. One case caused by *Trichophyton tonsurans* suffered subsequent herpes zoster infection, which shows that tinea capitis may be a risk factor for varicella zoster virus reactivation.^16^

## 4 CONCLUSION

Herein, we report a case of tinea capitis in a 75‐year‐old immunocompetent female and review the literature on this rare entity from 2018 to 2023. Despite the rarity of the disease in adults, tinea capitis should be included in the differential diagnosis of the inflammatory scalp lesions in adult or elderly patients, even in immunocompetent individuals. A dermoscopy and a KOH examination (and/or fungal culture) should be performed, to provide early and accurate treatment to minimize complications and sequelae of the disease.

## AUTHOR CONTRIBUTIONS

**Mehdi Gheisari:** Data curation; methodology; resources; supervision; validation; visualization; writing – original draft; writing – review and editing. **Khatere Zahedi:** Conceptualization; data curation; formal analysis; methodology; resources; writing – original draft; writing – review and editing. **Nabaa Al‐Zubaidi:** Data curation; formal analysis; methodology; project administration; software; writing – original draft; writing – review and editing.

## FUNDING INFORMATION

There was no funding provided for this case report.

## CONFLICT OF INTEREST STATEMENT

The authors confirm that there is no conflict of interest.

## CONSENT

In compliance with the journal's patient consent policy, the patient's written informed consent was acquired for the publication of this report.

## Data Availability

The data are available from the corresponding author upon reasonable request.

## References

[1] Hay RJ . Tinea capitis: current status. Mycopathologia. 2017;182(1–2):87‐93. doi:10.1007/s11046-016-0058-8 27599708 PMC5283510

[2] Ahmad S , Wani Ghm , Khursheed B . Kerion mimicking bacterial infection in an elderly patient. Indian Dermatol Online J. 2014;5(4):494‐496. doi:10.4103/2229-5178.142518 25396139 PMC4228651

[3] Aguirre Sotelo JP , Tarango Martinez VM , Vera CL . Kerion celsi caused by *Trichophyton tonsurans* in an adult. An Bras Dermatol. 2022;97(5):637‐640. doi:10.1016/j.abd.2021.10.005 35850939 PMC9453518

[4] Lova‐Navarro M , Gómez‐Moyano E , Martínez Pilar L , et al. Tinea capitis in adults in southern Spain. A 17‐year epidemiological study. Rev Iberoam Micol. 2016;33(2):110‐113. doi:10.1016/j.riam.2015.02.007 26774593

[5] Grimalt R . Management of tinea capitis in childhood. Clin Cosmet Investig Dermatol. 2010;3:89. doi:10.2147/ccid.s7992 PMC3047946 21437064

[6] Grijsen ML , de Vries HJC . Kerion. CMAJ. 2017;189(20):E725. doi:10.1503/cmaj.160665 28536129 PMC5436964

[7] Veasey JV , Souza G , De MC . Tinea capitis: correlation of clinical presentations to agents identified in mycological culture. An Bras Dermatol. 2018;93(3):465‐466. 29924231 10.1590/abd1806-4841.20187435 PMC6001095

[8] Gupta AK , Friedlander SF , Simkovich AJ . Tinea capitis: an update. Pediatr Dermatol. 2022;39(2):167‐172. doi:10.1111/pde.14925 35075666

[9] Rothman S , Smiljanic A . The spontaneous cure of tinea capitis in puberty. J Invest Dermatol. 1947;8(2):81‐98. doi:10.1038/jid.1947.15 20286535

[10] Buckley DA . Lesson of the week: tinea capitis in adults. BMJ. 2000;320(7246):1389‐1390. doi:10.1136/bmj.320.7246.1389 10818032 PMC1118052

[11] Khurana A , Agarwal A , Agrawal D , et al. Effect of different itraconazole dosing regimens on cure rates, treatment duration, safety, and relapse rates in adult patients with tinea Corporis/Cruris: a randomized clinical trial. JAMA Dermatol. 2022;158(11):1269‐1278. doi:10.1001/jamadermatol.2022.3745 36103158 PMC9475442

[12] Sacheli R , Hayette MP . Antifungal resistance in dermatophytes: genetic considerations, clinical presentations and alternative therapies. J Fungi. 2021;7(11):983‐1006. doi:10.3390/jof7110983 PMC8622014 34829270

[13] Gu D , Hatch M , Ghannoum M , Elewski BE . Treatment‐resistant dermatophytosis: a representative case highlighting an emerging public health threat. JAAD Case Rep. 2020;6(11):1153‐1155. doi:10.1016/j.jdcr.2020.05.025 33134459 PMC7591325

[14] Saunte DML , Hare RK , Jørgensen KM , et al. Emerging terbinafine resistance in trichophyton: clinical characteristics, squalene epoxidase gene mutations, and a reliable EUCAST method for detection. Antimicrob Agents Chemother. 2019;63(10):1‐9. 10.1128/AAC.01126-19 PMC6761549 31383665

[15] Do N , Notaro E , Schillhammer G , Colven R . Tinea capitis mimicking favus in rural Washington State. JAAD Case Rep. 2020;6(3):187‐188. doi:10.1016/j.jdcr.2019.12.013 32149173 PMC7033294

[16] Zhu Y , Niu X , Geng S , et al. A 50‐year history of tinea capitis. Mycopathologia. 2021;186(3):469‐474. doi:10.1007/s11046-021-00557-x 33961230

[17] Zheng YS , Zhou XY , Luo J , et al. Adult black dot tinea capitis caused by *Trichophyton tonsurans* complicated with herpes zoster. Chin Med J (Engl). 2020;133(1):91‐93. doi:10.1097/CM9.0000000000000567 31923110 PMC7028189

[18] Moqadam SD , Mofarrah R , Amiri KJ , Montazer F , Barqi A , Mofarrah R . Tinea capitismimicking alopecia areata. Our Dermatology Online. 2021;12(1):40‐43. doi:10.7241/ourd.20211.10

[19] Takaliuang DS . Tinea capitis in adolescent: A case report. Eduvest ‐ J Univers Stud. 2022;2(1):55‐63. doi:10.59188/eduvest.v2i1.333

[20] Goto Y , Suzuki T , Suzuki Y , et al. Trichophyton tonsurans‐induced kerion celsi with decreased defensin expression and paradoxically increased interleukin‐17Aproduction. J Dermatol. 2019;46(9):794‐797. doi:10.1111/1346-8138.15008 31294481

[21] Alves F , Batista M , Gonçalo M . Inflammatory tinea capitis mimicking erosivepustulosis of the scalp. Acta Med Port. 2019;32(11):733. doi:10.20344/amp.11588 31703188

[22] Starace M , Boling LB , Bruni F , et al. Telogen‐sparing arthroconidia involvement in anadult case of endothrix tinea capitis. J Egypt Women's Dermatologic Soc. 2022;19(3):210‐212. doi:10.4103/jewd.jewd_24_22

[23] Errichetti E , Buligan C . Tinea capitis in a healthy adult: An unexpected diagnosis made on dermoscopy. Dermatol Pract Concept. 2021;11(4):e2021083. doi:10.5826/dpc.1104a83 35024219 PMC8648417

[24] Rabhi F , Elinkichari D , Mtibaa L , Jemli B , Jaber K , Dhaoui MR . Inflammatory tinea capitis mimicking dissecting cellulitis in a healthy woman. Ski Appendage Disord. 2023:1‐4. doi:10.1159/000530498 PMC10688241 38045471

[25] Yadav A , Garg T , Saha B , Chander R , Nangia A . Tinea capitis masquerading discoidlupus erythematosus. Int J Trichology. 2020;12(3):144‐145. doi:10.4103/ijt.ijt_90_19 33223747 PMC7659738

